# Betaine Alters the Interplay of the Adenosine and *NO* Systems in the Control of Renal Regional Haemodynamics and Excretion in Diabetic Female Rats

**DOI:** 10.3390/ijms27094076

**Published:** 2026-05-02

**Authors:** Leszek Dobrowolski, Anna Volodymyrivna Monchakivska, Małgorzata Rogozińska, Konrad Kowalski, Marta Kuczeriszka

**Affiliations:** 1 Department of Renal and Body Fluid Physiology, Mossakowski Medical Research Institute, Polish Academy of Sciences, 5 A. Pawińskiego Street, 02-106 Warsaw, Poland; lesdobro@imdik.pan.pl (L.D.); annamonchakivska@gmail.com (A.V.M.); 2 Departments of Biochemistry, Educational and Scientific Centre, Institute of Biology and Medicine, Taras Shevchenko National University of Kyiv, 02000 Kyiv, Ukraine; 3 MASDIAG Ltd., 33 S. Żeromskiego Street, 01-882 Warsaw, Poland; malgorzata.rogozinska@masdiag.pl (M.R.); konrad.kowalski@masdiag.pl (K.K.)

**Keywords:** betaine, diabetes, female, nitric oxide, oxidative stress, renal microcirculation, theophylline, renal tubular transport

## Abstract

We showed recently that the adenosine system and nitric oxide (*NO*) can interact differently in the control of renal function in normoglycaemia (NG) versus streptozotocin-induced diabetes (DM). Herein, we investigated if this relationship is modulated by dietary betaine (Bet, food compound possessing antioxidant and anti-inflammatory properties), to examine if adenosine receptor signalling in NG and DM females is altered by chronic Bet supplementation. The effects of intravenous infusion of theophylline, non-selective adenosine receptor antagonist, were examined in anaesthetised Sprague–Dawley female rats, pretreated for 2 weeks with Bet alone or combined with 4-day *NO* synthesis blockade with L-NAME (Bet + L-NAME). Renal blood flow (RBF, ultrasound artery probe), perfusion of the cortex, outer (OM-BF) and inner medulla (IM-BF; laser-Doppler technique), and tissue *NO* signal (selective electrode) were determined along with renal excretion. Bet and Bet + L-NAME decreased baseline RBF irrespective of glycaemia, whereas Bet lowered (NG) or elevated (DM) basal OM-BF; Bet + L-NAME treatment abolished these effects. Baseline sodium excretion decreased after Bet and Bet + L-NAME in NG only. Bet modified theophylline effects: IM-BF was lowered in DM rats, while tissue *NO* changes shown in the control were modified: *NO* increased in NG and decreased in DM. In NG, these effects were abolished by Bet + L-NAME. Bet pretreatment did not alter diuresis, natriuresis and kaliuresis, but after Bet + L-NAME these parameters increased (NG) or decreased (DM). Dietary Bet has the potential to affect renal medullary blood circulation; however, the eventual effect depends on glycaemia. Bet can modify renal functional changes induced by the interplay of the adenosine and *NO* systems, both in rats with normoglycaemia and streptozotocin diabetes.

## 1. Introduction

In 2021, the global prevalence rate of *diabetes mellitus* (DM) was over 6%, i.e., among the top 10 causes of death [1]. This metabolic disease causes multiple morphological and functional changes in many organs, including the kidney. The basis of the associated pathophysiological changes is microvascular damage or microangiopathy, affecting both endothelial cells and smooth muscle cells; both interact in the regulation of blood flow [2].

The maintenance of vascular function by endothelial-derived *NO* is essential under physiological conditions. In the blood vessel wall, *NO* is mainly produced from l-arginine by endothelial *NO* synthase (eNOS), yet other mechanisms of vascular *NO* production exist [3].

The pathogenesis of DM involves oxidative stress (OS) and inflammation. Interestingly, it was demonstrated that modulation of inflammatory pathways is useful in diabetic retinopathy [4,5]. The former (i.e., OS) can be defined as an imbalance between oxidant production and antioxidant defence; this can harm biological systems, resulting in vascular damage [6]. OS is the cause of endothelial dysfunction (ED), the result of the imbalance between antioxidant defence mechanisms and the generation of reactive oxygen species (ROS). The molecular mechanisms responsible for ED are complex and exerted by various stimulants, including *NO*, low-density lipoprotein, ROS, shear stress, and high glucose [7]. Thus, ED is an important factor in the aetiology of DM, cardiovascular diseases (CVDs) and hypertension (HT).

ED has been implicated in both diabetic animal models and human patients. It is currently widely believed that excess ROS produced by mitochondrial and cytoplasmic oxidases (e.g., Nicotinamide adenine dinucleotide phosphate, NADPH oxidase) might trigger early stages of microvascular damage. Elevated levels of glucose or fatty acids can expedite this process.

Reduced bioavailability of endothelial NOS (eNOS)-derived *NO* is the primary sign of endothelial dysfunction, and endogenous eNOS activation in diabetes can be protective, e.g., against atherosclerosis [6]. It is important to maintain the physiological level of *NO*, in crucial tissues, mainly by preventing its over-synthesis (seen in early diabetes) and further rapid metabolism followed by ROS overproduction. Thereby, ROS-dependent negative effects leading to endothelial dysfunction are prevented.

Several studies have shown that markers of OS are elevated in patients with diabetes. A key consequence of OS is the reduction in the availability of *NO*, a critical molecule for maintaining vascular homeostasis through vasodilation. With the growing understanding of the basic pathophysiology of diabetes, it becomes obvious that oxidative stress and nitrosative redox alterations are key factors in DM pathogenesis and its complications [8].

*NO* is essential for regulation of kidney vascular tone [9,10]; inhibiting its synthesis leads to increased renal vascular resistance (RVR), reduced renal plasma flow and decreased sodium excretion (U_Na_V) and can ultimately cause HT [11]. An optimal ROS production is essential for normal kidney function: excessively high levels of ROS can cause renal vasoconstriction and damage to cells. In the kidney, ROS and *NO* interact to regulate fluid and electrolyte balance, playing a key role in solute and water reabsorption to maintain electrolyte and extracellular fluid volume homeostasis [12,13].

While *NO* has a natriuretic and diuretic action by inhibiting sodium transport [13], ROS are crucial second messengers for cell signalling, promoting normal cell functions like growth and causing inflammation. However, excessive amounts of ROS can constrict renal vessels by changing the effect of the factors released by the endothelium.

It should be noticed here that NOS activity is sex-dependent: it is higher in females than in males, and the result might be greater *NO* synthesis in the former, possibly triggering excessive synthesis of ROS [14].

In the renal medulla, the balance between *NO* and ROS generation is crucial for regulation of medullary perfusion (MBF) and U_Na_V [15]. Elevated medullary superoxide (O_2_^−^) and derivative molecules, such as hydrogen peroxide (H_2_O_2_), an endogenous ROS, reduce MBF and might promote sodium reabsorption, leading to HT, while *NO* has the opposite, protective effect. Increases in renal perfusion pressure elevate both medullary H_2_O_2_ and *NO*, but the excess H_2_O_2_ can impair the normal increase in *NO*, disrupting the balance and potentially contributing to HT [16]. Thus, the NO/ROS balance is crucial for physiological vascular state (dilation and constriction) and might be often disturbed in diabetes or HT. New measures to preserve the balance are being looked for.

Vast research has been conducted on compounds of natural origin with strong antioxidant properties, mostly on vitamins and flavonoids of plant origin, in hopes of making use of their antioxidative properties in patients with CVD. Antioxidant therapy shows promise for preventing and treating CVD, and vitamins are the group with the strongest antioxidant potential, especially vitamins A and E (lipid-soluble) and C (water-soluble) [17].

Many studies have attempted to explain the possible mechanisms whereby vitamin A (retinol, Vit. A) affects diabetes development [18]. It influences cell development, including pancreatic cells, and thereby could be involved in insulin production [19,20]. Vit. A activates transcriptional networks controlled by retinoic acid receptors: this helps diabetic patients reduce the level of free radicals in the body and thus attenuate inflammation [21].

The Vit. E group consists of lipophilic antioxidants that include both classes of organic compounds: tocopherols and tocotrienols [22]. Vit. E, a putative radical scavenger, is probably the most important inhibitor of radical-induced lipoprotein lipid peroxidation [23,24]. In mice, α-tocopherol inhibits atherogenesis and improves cardiac function independently of its antioxidant properties. On the other hand, negative correlation was widely described between these vitamins’ blood levels and the likelihood of CVD development [25]. Furthermore, the basic tissue and plasma antioxidant vitamin levels might reflect the oxidative status of the animal.

Profound studies are conducted with Bet (trimethylglycine), a naturally occurring compound found in animals, plants, and microorganisms. It is synthesised endogenously and consumed through dietary intake [26] and possesses antioxidant and anti-inflammatory properties [27,28]. Its endogenous synthesis cannot fulfil the body requirement, and dietary supplementation might sustain or improve health [29].

Notably, Pelpolage et al. [30] reported that dietary intake of Bet might improve serum *NO* bioavailability in both normotensive and hypertensive rats [30]. Furthermore, the results obtained by Sun et al. [31] showed that Bet is able to repair microvascular endothelial damage, which was stopped by L-NAME (non-selective NOS inhibitor) delivery, suggesting *NO*/NOS involvement within this process [31].

In the kidney, Bet protects the cells from osmotic stress. It counteracts the elevation of osmotic pressure outside the cell, thereby preserving the cell’s normal volume [32].

Adenosine (Ado) in the renal vascular bed can induce both vasoconstriction (via P1-A1 receptors) and vasodilation (via P1-A2 receptors), with the latter often mediated by *NO* [33]. A typical pattern of P1 receptor (P1R) distribution in the kidney—more P1R-A1 in the cortex and more P1R-A2 in the medulla—can be reversed in diabetes [34,35,36]. P1R-A2 promotes natriuresis (inhibits sodium reabsorption), while P1R-A1 has antinatriuretic (sodium-retaining) effects.

P1R is involved in regulation of glucose metabolism in diabetes, with adenosine signalling via a pathway that elevates intracellular cyclic adenosine monophosphate (cAMP) to increase glucose uptake [37].

As mentioned above, Bet acts as an osmolyte accumulated by renal medullary cells to sustain outside/inside osmotic balance. Ado has been shown to acutely inhibit betaine transport in canine kidney cells (MDCK), a model used frequently to study P1R activity and impact renal tubular transport [38,39].

On the other hand, it is important to indicate that there exists a close interrelation- ship between *NO* and Ado receptors both in NG and DM rats (adenosine-caused vaso- dilation seems to be *NO*-dependent) [40].

In this project, we investigated the effects of Bet supplementation in the streptozotocin (STZ)-induced diabetes rat model, to determine if it would improve kidney function and/or modulate the levels of antioxidant vitamins (retinol and α-tocopherol) in the blood, kidney, and liver tissues. The project involves inducing diabetes in rats using STZ (damages pancreatic ß-cells) and then assessing the impact of chronic Bet supplementation on renal function and the concentrations of these specific vitamins.

The focus was to examine if the effect of P1R signalling on renal function and *NO* content in the rat in situ kidney differs between NG and DM females with or without chronic betaine supplementation. To this purpose, the impact of a non-selective P1R-A1 and P1R-A2 antagonist theophylline (Theo) was determined in anaesthetised rats that were either normoglycaemic or in the state of hyperglycaemia (experimental DM) induced two weeks earlier with STZ. We hypothesised that the impact of Bet on P1R signalling differs between the renal cortex and medulla because perfusion of the latter seems especially sensitive to *NO* and ROS imbalance [41]. For a more detailed insight, blood circulation of the outer and inner medulla was determined separately. Based on the mentioned interaction of adenosine and *NO*, we also explored the role of *NO* in the effects of Theo in rats co-treated with Bet and L-NAME, a NOS inhibitor.

## 2. Results

### 2.1. Chronic Study

During the chronic part of the study, several parameters were tested. For ease and clarity of presentation, we have decided to show only those that show significant changes or differences. In Table 1 the data cumulated over the period of two weeks study with conscious animals, i.e., after injection of buffer or STZ (NG or DM rats, respectively), supplemented with Bet alone for the 14 days (left side in Table 1) or with Bet + L-NAME co-treatment for the last four days (right side in Table 1) before the acute experiment, are shown. The non-significant data are shown in the Supplementary File S2 (Table S1).

#### 2.1.1. Phase of the Oestrus Cycle

To determine the oestrous cycle phase, a vaginal smear was performed. The proestrus phase was determined in all female rats. This was possible due to good synchronisation of the animals: rats were placed in the same experimental group before the start of the experiment.

#### 2.1.2. Effects of Bet and L-NAME Pretreatments on Body Weight and Blood Glucose Level

In NG females, there were no distinct body weight (BWt) changes during 14 days of observation, independent of treatment. In non-pretreated diabetic females, BWt did not differ between day 0 and 14 (260 ± 7 vs. 263 ± 5 g), whereas after treatment with Bet alone or with NOS inhibitor (Bet + L-NAME_4_), a BWt decrease was observed.

Irrespective of the chronic treatment with Bet without or with L-NAME_4_, in all three normoglycaemic experimental groups (NG, as well NG + Bet_14_ and NG + Bet_14_ + L-NAME_4_), blood glucose (BG) remained stable (see Table 1). As could be predicted, in DM rats BG was higher beginning from the third day after STZ injection (above 400 mg/dL) and remained elevated until the end of the observation during the chronic part of the study. L-NAME_4_ treatment did not alter BG in either NG or DM rats.

#### 2.1.3. Effects of Bet and L-NAME Pretreatments on Blood Parameters

The haematocrit (Hct) levels also remained unchanged in NG independent of treatments, in all three experimental groups (see Table 1). However, in the DM + Bet (alone) group an increase was shown, while the addition of L-NAME to drinking water (DM + Bet + L-NAME_4_) did not alter Hct.

#### 2.1.4. Effects of Bet and L-NAME Pretreatments on Daily Water/Food Intake and Urine Excretion

Daily water intake was unchanged in NG rats both by treatment with Bet alone and in combination with L-NAME; however, in DM rats the treatment with Bet alone was associated with increased water intake (about fourfold). Simultaneously, food intake increased at least twice. Daily urine excretion decreased in both NG + Bet_14_ and NG + Bet_14_ + L-NAME_4_ rats and this was associated with urine osmolality (U_osm_) increase, whereas in DM rats Bet treatment caused an increase in diuresis in parallel with a U_osm_ decrease. However, in the DM + Bet_14_ + L-NAME_4_ group, although Bet treatment increased urine excretion (not modified by L-NAME addition), U_osm_ was not altered significantly.

### 2.2. Acute Experiments

#### 2.2.1. Effects of Bet and L-NAME Pretreatments in NG and DM Rats on Baseline Haemodynamics, Renal Circulation and Tissue *NO*

In Table 2, the mean values of haemodynamics, renal perfusion and excretion from the last two control periods (basal values 30 min before infusion of Theo given intravenously, (*i.v.*) are collected and analysed. Without any treatment, no difference in mean blood pressure (MBP) between NG and DM groups was observed during control periods. In both NG and DM groups, pretreatment with Bet only virtually did not alter basal MBP but, as could be expected, the level was increased by L-NAME_4_ treatment (Bet + L-NAME_4_): in NG by 10% (*p* < 0.001), and in DM by 13% (*p* < 0.01). Noteworthily, the basal heart rate (HR) (higher in non-pretreated NG than DM females by about 40 beats/min, *p* < 0.01) was not modified by Bet pretreatment, whereas after Bet and L-NAME_4_ co-treatment HR was lower independent of glycaemia, and, again, it differed between NG and DM (by about 65 beats/min, *p* < 0.01).

The reason for *n* = 3 only (DM + Bet + L-NAME group) was that after chronic observations, some rats under this specific pretreatment were highly sensitive to anaesthesia and extensive surgery of acute experiments. They died in their initial phase or even during surgical preparation, so only the results of the full-time acute experiments are shown. We believe that increasing the number of animals would not have yielded clearer results, given the discomfort they experienced.

We would like to emphasize that, initially, in animals with this specific chronic pre-treatment (L-NAME), we reduced the dose of anaesthesia (thiopental); however, even this did not give a positive result. A similar situation (hypersensitivity to anaesthesia) occurred in our earlier study with rats maintained on a high-sodium diet (4% Na *w*/*w*) for 21 days and subjected to analogous chronic blockade of NOS activity by administering L-NAME solution to drink for the last 4 days before the acute experiment. In those rats, reducing the anaesthetic dose was effective [40].

Basal RBF and cortical blood flow (CBF) were significantly higher in NG than in DM rats (*p* < 0.01 and *p* < 0.0003, respectively). Bet pretreatment lowered basal RBF independently of glycaemia, and as expected, L-NAME_4_ pretreatment (Bet + L-NAME_4_) further lowered RBF in both NG and DM groups (by 23%, *p* < 0.03 and 39%, *p* < 0.01, respectively). Unlike with RBF, Bet lowered baseline CBF significantly in a glycaemia-dependent manner (i.e., in NG group only), whereas L-NAME_4_ co-treatment did not alter CBF independent of glycaemia.

While basal mean values for OM-BF and IM-BF, similarly as for RBF and CBF, were higher in NG than in the DM group, the differences between groups were not significant. Interestingly, in Bet-pretreated rats OM-BF was lower (*p* < 0.02) in NG, whereas in the DM group it was higher (*p* < 0.0001) than in their non-pretreated counterparts. L-NAME treatment restored outer medulla perfusion close to the values shown in NG and DM rats without any treatment. Unlike with OM-BF, neither Bet alone nor Bet + L-NAME_4_ pretreatment significantly changed basal IM-BF.

Notably, similar to basal OM-BF, baseline tissue *NO* signal values also did not differ between normo- and hyperglycaemic females; after Bet treatment, it was similar in NG but noticeably higher (*p* < 0.02) in the DM group compared with the non-pretreated counterparts (Table 3). Furthermore, after L-NAME co-treatment, tissue *NO* in DM was shown to be restored, whilst in NG group it was elevated (*p* < 0.03).

It should be clearly pointed out that our *NO* measurement method was applied to estimate changes in tissue *NO* bioavailability over the course of a given experiment; the respective analysis is described below (see Section 2.2.5). On the other hand, the method may not be quite suitable for a reliable comparison of basal *NO* signal values between groups.

However, during analysis of the results of the present experimental set-up, we noticed quantitative differences in baseline NO signal values between groups. Interestingly, these differences clearly accord with those of basal perfusion of the outer medulla (OM-BF).

Admittedly, the above analysis is just an accidental finding, but it can engender further systematic research to overcome limitations of the amperometric measurement of tissue *NO* signal.

#### 2.2.2. Effects of Bet and L-NAME_4_ Pretreatment in NG and DM Rats on Baseline Renal Excretion

There was a baseline urine flow (V) difference between untreated NG and DM rats (*p* < 0.03). Bet pretreatment slightly reduced baseline diuresis in NG rats in comparison with the non-pretreated group, whereas no Bet effect on urine flow (V) was shown in DM rats. Ultimately, baseline diuresis did not differ between the Bet-treated NG and DM groups. L-NAME added to Bet treatment did not distinctly alter V, similar to results in NG and DM rats.

There was a baseline U_osm_ difference between untreated NG and DM rats (775 ± 70 vs. 1020 ± 20 mosmol/kg H_2_O, respectively, *p* < 0.03). Bet pretreatment did not alter U_osm_ in NG rats, whereas in DM females there was a change but it was not significant. Again, baseline U_osm_ did differ between Bet-pretreated NG and DM animals (780 ± 34 vs. 880 ± 74 mosmol/kg H_2_O, respectively, *p* < 0.04). After L-NAME addition to Bet treatment, U_osm_ did not differ between NG and DM groups (905 ± 66 vs. 985 ± 60 mosmol/kg H_2_O, respectively, NS).

There was no baseline of urine total solutes excretion (U_osm_V) difference between NG and DM rats and this was not modified by Bet pretreatment alone. However, in the NG group with additional L-NAME pretreatment (Bet + L-NAME_4_), U_osm_V was significantly lower than in rats without any treatment (*p* < 0.03). Notably, in DM rats the differences between untreated and pretreated rats were not significant.

It is noteworthy that U_Na_V was significantly higher in NG than in DM females (*p* < 0.02). After Bet pretreatment, this difference disappeared. In NG rats, U_Na_V was lower (*p* < 0.02), whereas in DM it was similar to the values in their non-pretreated counterparts. Such relationships were also not altered when L-NAME_4_ was added to Bet pretreatment. Again, U_Na_V was lower in NG vs. non-pretreated animals (*p* < 0.01), it did not change significantly in DM rats, and did not differ between NG and DM groups.

Baseline urine potassium excretion (U_K_V) was significantly higher in NG than in DM females (*p* < 0.0005). This difference disappeared in Bet-treated rats; however, independent of glycaemia, U_K_V was significantly lower than in their non-pretreated counterparts (*p* < 0.001 and *p* < 0.01, for NG and DM rats, respectively). Notably, after co-treatment (L-NAME_4_ plus Bet) U_K_V did not change significantly in NG, whereas in the DM group it increased compared with the animals treated with Bet alone (*p* < 0.005).

#### 2.2.3. Impact of Theophylline on Blood Pressure, Heart Rate, and on Whole Kidney and Regional Blood Perfusion in NG and DM Rats Pretreated with Bet and L-NAME

**Figure 1:**
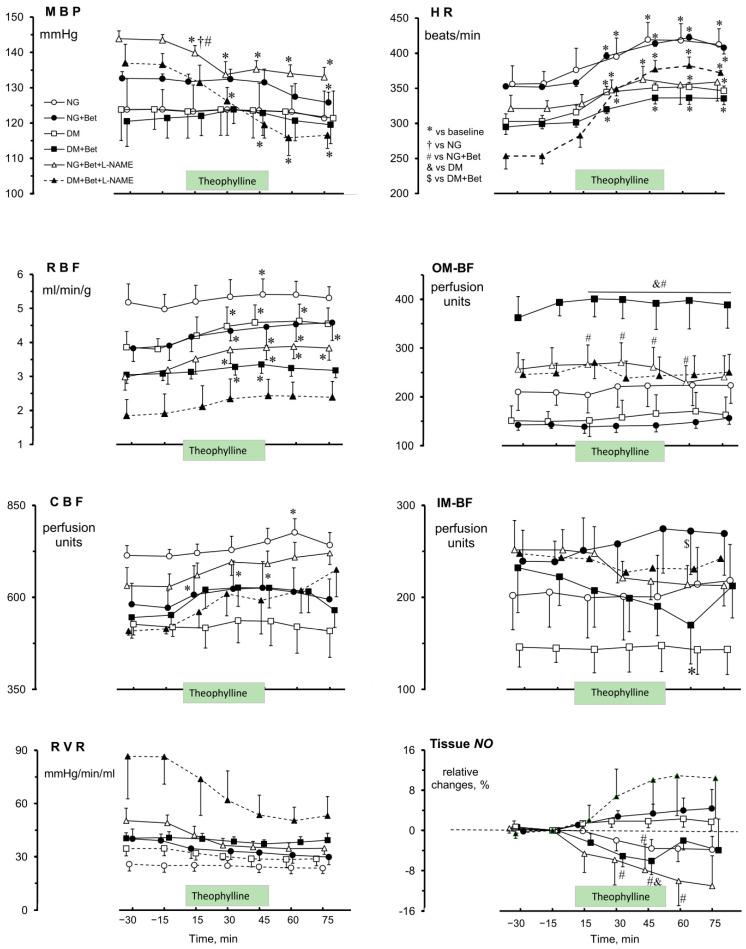
Effects of theophylline on MBP, HR, renal haemodynamics and in situ tissue *NO* signal in the renal medulla of normoglycaemic (NG) and hyperglycaemic female rats (DM), non-pretreated or pretreated with Bet (betaine alone) or combined with L-NAME (Bet + L-NAME). Green box on the time axis indicates theophylline infusion period preceded and followed by solvent infusion. Means ± SEM; *n* = 6–8, each group with the exception of DM Bet + L-NAME, *n* = 3 (time-course marked with dashed line). MBP—mean blood pressure; HR—heart rate; RBF—whole kidney blood flow; CBF, OM-BF and IM-BF—cortical, outer and inner medullary blood flow, respectively; RVR—renal vascular resistance. * different vs. the respective baseline; † different vs. the NG non-pretreated rats; # different vs. NG rats pretreated with Bet; & different vs. DM non-pretreated rats; $ different vs. DM rats pretreated with Bet.

It is noteworthy that both in NG and DM rats blood pressure remained unaffected by *i.v* Theo, both in rats pretreated with Bet and in their non-pretreated counterparts. Remarkably, in the Bet + L-NAME_4_ co-pretreated groups, independent of glycaemia, Theo caused an MBP decrease (by 10–17 mmHg, *p* < 0.03) without recovery after cessation of the drug infusion (Figure 1, MBP). Interestingly, the post-Theo increase of HR (by 18–19%) did not differ between NG and DM females, whereas in those after Bet pretreatment it was higher in NG than in DM rats (20 ± 2% vs. 12 ± 1, respectively, *p* < 0.001). Meanwhile inverse changes were shown after Bet + L-NAME_4_ treatment: the Theo-induced HR increase was lower (and transient) in NG than in DM animals (14 ± 5% vs. 45 ± 10%, respectively, *p* < 0.001) (Figure 1, HR).

After Theo infusion in rats without any pretreatment, an increase in RBF was shown in both normo- and hyperglycaemic rats (13 ± 4% and 25 ± 9%, respectively, *p* < 0.03), but in the former only transiently. However, after pretreatment with Bet, RBF was increased by Theo (17 ± 5%) in NG females (*p* < 0.03) without recovery after cessation of drug infusion, whereas in DM animals the increase was smaller (12 ± 3%, *p* < 0.02) and transient. Interestingly, in L-NAME pretreated groups (Bet + L-NAME_4_), Theo caused a gradual RBF increase in NG rats (by 35 ± 5%, *p* < 0.001) without recovery after cessation of drug infusion, whereas in DM rats the changes were not significant (Figure 1, RBF). On the other hand, independent of any pretreatment, Theo did alter CBF only in NG rats (by 11–15%, *p* < 0.03) whereas no significant changes were shown in the DM group (Figure 1, CBF).

The medullary blood flow changes were different from those seen in the cortex. Medullary blood perfusion of the inner zone was altered by Theo only in DM rats pretreated with Bet where a decrease in IM-BF was shown (12 ± 3%, *p* < 0.01) (Figure 1, OM-BF, IM-BF).

Since in NG and DM rats RBF increases caused by Theo arose without any significant changes in MBP, the calculated RVR did not alter in normo- and hyperglycaemic rats and usually kept close to the basal value even after interruption of drug infusion. This was not affected by Bet pretreatment, and Theo did not induce any changes. Dissimilarly, in those rats pretreated with L-NAME_4_ Theo did evoke a visible RVR decrease and no recovery to the basal value until the experiment was terminated (Figure 1, RVR). However, these changes did not reach statistical significance.

#### 2.2.4. Impact of Theophylline on Renal Excretion in Normo- and Hyperglycaemic Rats Bet and L-NAME Pretreated

**Figure 2:**
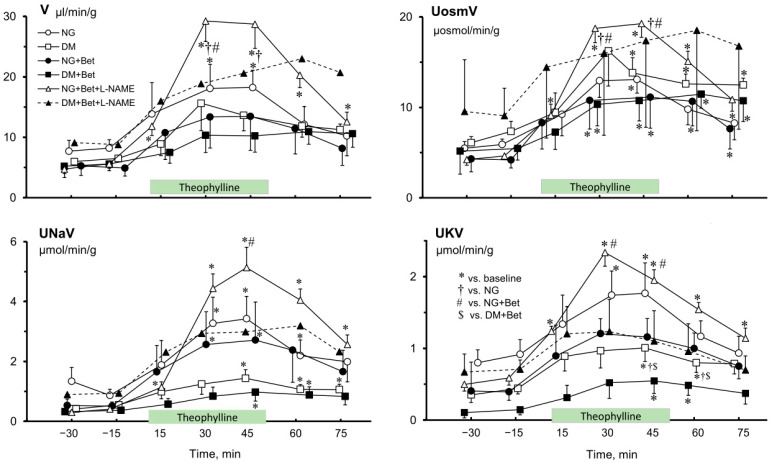
Effects of theophylline on renal excretion in normoglycaemic (NG) and hyperglycaemic female rats (DM), untreated or pretreated with betaine alone (Bet) or combined with L-NAME (Bet + L-NAME). Green box on the time axis indicates theophylline infusion period preceded and followed by solvent infusion. Means ± SEM; *n* = 6–8, each group with exception of DM Bet + L-NAME, *n* = 3 (time-course marked with dashed line). V—urine flow; U_osm_V, U_Na_V, U_K_V—total solute, sodium and potassium excretion, respectively. * different vs. the respective baseline; † different vs. the non-pretreated NG rats; # different vs. NG rats pretreated with Bet; $ different vs. DM rats pretreated with Bet.

Similarly to the impact of Theo on renal blood perfusion (Figure 1), the changes induced to parameters of renal excretion were unidirectional (Figure 2). Usually an increase of V, U_osm_V, U_Na_V and U_K_V during Theo infusion was seen in NG and DM rats irrespective of Bet or Bet + L-NAME_4_ pretreatment. The exception was U_osm_ which in NG rats was also elevated after Theo infusion, in untreated and even more so in Bet-pretreated rats as compared with respective baseline values (775 ± 70 vs. 900 ± 85, *p* < 0.03 and 780 ± 35 vs. 1135 ± 150 mosmol/kg H_2_O, *p* < 0.04, respectively). Surprisingly, Theo induced a U_osm_ decrease in Bet + L-NAME_4_ pretreated animals (905 ± 65 vs. 680 ± 70 mosmol/kg H_2_O, *p* < 0.01). On the other hand, U_osm_ did not change in DM animals without any pretreatment, it increased in those with Bet-alone treatment (880 ± 75 vs. 1105 ± 105 mosmol/kg H_2_O, *p* < 0.04), whereas no changes were seen in rats pretreated with Bet + L-NAME_4_.

V was increased by Theo in NG rats, as compared with the respective baseline, similarly in those without and with Bet pretreatment (by 153 ± 35 vs. 159 ± 36%, respectively, *p* < 0.01), and clearly more so in the Bet + L-NAME_4_-pretreated rats (658 ± 179%, at *p* < 0.01 vs. baseline or *p* < 0.03 vs. Bet-alone pretreatment). On the other hand, in DM rats Theo-induced V elevation was less distinct in both non-pretreated and Bet-pretreated groups (114 ± 50% and 115 ± 26%, respectively, *p* < 0.01) or even not significant in the Bet + L-NAME_4_-pretreated group (135 ± 94%, NS) (Figure 2, V).

As also seen with V, Theo increased U_Na_V in NG and NG + Bet groups by 320 ± 70% and 435 ± 85%, respectively (*p* < 0.01 vs. corresponding baseline) and did so significantly more after co-pretreatment with Bet and L-NAME_4_ by 1575 ± 345% (*p* < 0.004 vs. baseline and *p* < 0.02 vs. Bet-alone pretreatment). On the other hand, in DM rats Theo did induce significant changes in sodium excretion in non-treated rats by 190 ± 55% (*p* < 0.03) and in Bet-alone-pretreated (140 ± 20%, *p* < 0.001) but not in Bet + L-NAME_4_-pretreated groups (220 ± 105%, NS) (Figure 2, U_Na_V).

The increase in potassium excretion (U_K_V) caused by Theo in NG rats proved lower in non-pretreated rats compared with those co-pretreated with Bet and L-NAME_4_ counterparts (90 ± 30%, *p* < 0.02 and 325 ± 110%, *p* < 0.02, respectively, at *p* < 0.05 between groups). On the other hand, in DM rats the mean post-Theo U_K_V increase was greater in non-treated rats than in the Bet + L-NAME_4_-pretreated group by 250 ± 20% (*p* < 0.04) vs. 175 ± 35% (NS), respectively. In the group pretreated with Bet alone, U_K_V elevation was visibly higher (525 ± 175, *p* < 0.04) than in the group without any pretreatment; however, the difference was not statistically significant (*p* < 0.06) (Figure 2, U_K_V).

A similar tendency to post-Theo changes was seen for U_osm_V in NG rats in which the excretion rate was lower in non-treated rats vs. counterparts co-pretreated with Bet and L-NAME_4_ (140 ± 30%, *p* < 0.01 and 310 ± 30%, *p* < 0.0004, respectively, at *p* < 0.01 between groups). However, in DM rats, both in non-pretreated rats and those co-pretreated with Bet + L-NAME_4_, U_osm_V was not significantly affected whereas in those pretreated with Bet alone a marked post-Theo increase was shown (by 150 ± 35%, *p* < 0.01) (Figure 2, U_osm_V).

#### 2.2.5. Effects of Theophylline on Renal Tissue *NO* in NG and DM Females Pretreated with Bet and L-NAME

The renal medullary tissue *NO* signal after Theo infusion was lowered slightly or increased somewhat compared to the baseline level in non-pretreated NG versus DM animals, respectively, but the difference between groups was not significant (Figure 1, tissue *NO*). Bet pretreatment reversed the Theo effect: a distinct tissue *NO* increase or decrease was shown in NG and DM, respectively (*p* < 0.02). This effect was clearly altered by L-NAME co-treatment; again, the Theo-induced decrease in NG rats significantly differed from the change (an increase) described above in Bet-pretreated counterparts (*p* < 0.04) (Figure 1). On the other hand, in DM rats the mean value after Theo was above baseline but not significantly different compared with the changes seen after pretreatment with Bet alone.

### 2.3. Serum and Tissue Vitamins

The selection of tissues for measurement of vitamins with antioxidant potential (retinol and tocopherol) was done considering their physiological concentrations (N.B.—in mammals, the liver is the richest source of Vit. A and E).

#### 2.3.1. Effects of Bet and L-NAME Pretreatments on Serum Vitamin

Serum retinol concentration was significantly lower in DM than in NG rats (128 ± 5 vs. 144 ± 3 ng/mL, *p* < 0.05, respectively). Chronic oral treatment with Bet had no impact on plasma retinol in NG females, whereas increased concentration was shown in DM rats (164 ± 11 vs. 128 ± 5 ng/mL, *p* < 0.05, Bet-treated vs. Bet non-treated, respectively).

Serum α-tocopherol concentration was significantly higher in DM than in NG rats (11,029 ± 647 vs. 8467 ± 619 ng/mL, *p* < 0.05, respectively). Chronic treatment with Bet increased serum α-tocopherol in NG females as compared with those untreated (12,731 ± 773 vs. 8467 ± 619 ng/mL, *p* < 0.05, respectively), whereas decreased vitamin concentration was measured in DM rats (9298 ± 819 vs.11,029 ± 647 ng/mL, *p* < 0.05, with Bet vs. without Bet treatment, respectively).

#### 2.3.2. Effects of Bet Pretreatments on Renal and Liver Tissue Vitamin

Tissue vitamin data are presented in Supplementary File S2 (Supplementary Data: Vitamins concentration in renal and hepatic tissue) in Table S2A (retinol) and Table S2B (α-tocopherol). The renal tissue retinol concentration was about 30% higher in NG rats compared with those who drank Bet, whereas it did not differ from those shown in DM females, both with and without Bet pretreatment.

In hepatic tissue, retinol concentration was higher in NG than DM rats whereas chronic pretreatment with Bet did not affect vitamin concentration in NG but increased it in DM females to a level not different from that shown in NG rats.

The renal tissue α-tocopherol concentration did not differ between NG and DM, similarly with and without Bet pretreatment. Unlike with retinol, the hepatic tissue concentration of α-tocopherol was significantly (twice) lower in NG than in DM rats. Noteworthily, in DM rats chronically treated with Bet we saw a significantly lower vitamin concentration as compared with untreated DM rats; in the latter, it did not differ from the level measured in NG.

## 3. Discussion

(1) We studied the effects of chronic betaine treatment on the role of the endogenous adenosine system and its interaction with *NO* in controlling renal and systemic circulation and renal excretion, and to compare these effects in female rats without and with streptozotocin-induced diabetes.

Of note, sex-dependent changes were not the subject of the study; females were selected mechanistically, as the higher NOS activity described in them caused more pronounced changes in the amount of ROS, which could be additionally intensified by the initial phase of diabetes.

(2) In addition, we compared these relationships in diabetic and age-matched normoglycaemic rats, and between animals supplemented with betaine alone or combined with *NO* synthase inhibitor.

(3) We postulated that in the kidney the effects of endogenous adenosine on circulation and excretion are modified by betaine supplementation and depend on the actual experimental glycaemia status.

To eliminate any potential influence of the oestrous cycle on the results, the female animals were assorted in appropriately synchronised groups. Such synchronisation of the oestrus cycle ensured a similar internal environment and made the results obtained much more reliable.

Discussed below are the data from female Sprague–Dawley rats (SD) supplemented with betaine along the chronic part of the study and in betaine-treated animals also receiving the *NO* synthase inhibitor (NOSI). The results are compared with those obtained earlier in untreated females (i.e., drinking tap water) or those obtained in animals treated with NOSI alone [40].

### 3.1. Differences Between NG and DM Rats Treated with Bet

#### 3.1.1. Chronic Observations

Hyperglycaemic rats showed a decrease in BWt, irrespective of changes in food intake and faeces excretion, possibly depending on the STZ-induced hypoinsulinaemia. The contribution to the BWt decrease of dehydration secondary to osmotic diuresis is less likely.

#### 3.1.2. Comparison of Serum and Tissue Vitamin Levels Between NG and DM After Treatment with Bet

Serum Vit. A level was significantly higher in NG than in DM rats. Notably, an inverse relationship was reported between the retinol level and the development of diabetes; however, such data were inconsistent [42]. In our study, the between-group difference of Vit. A level was seen in the liver but not in the kidney tissue. The storage and re-mobilisation of retinol in the liver were reported to determine the serum concentrations of Vit. A [42]. Possibly, these processes are not crucial in the kidney and renal tissue vitamin A level is not dependent on actual blood glucose status.

Somewhat surprisingly, two weeks of exposure to Bet (in the diet) caused changes in serum and liver vitamin levels in DM, whereas in the kidney it did so in the NG group only. Presumably, Bet helps diabetic rats restore Vitamin A and its antioxidant function, and thereby could directly and indirectly reduce the level of free radicals in the body and thus relieve hyperglycaemia-dependent inflammation [21].

Most recently, serum Vit. E was reported to be significantly lower in DM compared to NG patients (Bangladeshi population) [43], unlike in our female SD rats. Another study showed that Vit. E deficit was associated with about a 4-fold increase in the chance of DM development [44]. On the other hand, DM patients (Mexican population) showed higher levels of Vit. E compared to healthy individuals; increased levels of Vit. E were proposed to be a protective factor [45].

In our study, unlike Vit. A, serum Vit. E was significantly lower in NG than in DM rats. Again, the same between-group difference of Vit. E level was shown in the liver but not in kidney tissue. Apparently, as with Vit. A, in the kidney the mechanism responsible for tissue vitamin E level is less affected by hyperglycaemia than in the liver.

Interestingly, in serum but not in liver tissue Bet treatment reversed the difference in Vit. E level between NG and DM rats. Serum Vit. E levels were elevated in normo- and reduced in hyperglycaemic female rats, whereas in the liver tissue Bet-pretreatment reduced Vit. E in hyperglycaemic but not in normoglycaemic animals. Possibly, in diabetes an elevated Vit. E level could protect against oxidative stress, and Bet, as mentioned above, could directly and indirectly reduce the level of free radicals in the body. Thus, lowering Vitamin E concentration after Bet treatment was sufficient to prevent oxidative stress.

Admittedly, the vitamin level was here measured in whole kidney tissue whereas Bet-treated diabetic rats showed distinct changes in medullary blood perfusion (Figure 2, OM-BF and IM-BF), without any differences in whole kidney blood flow (Figure 2, RBF). Thus, our whole kidney vitamin data could not reflect possible alterations in a relatively small part of the organ (medulla).

As was mentioned earlier, the tissue and plasma levels of antioxidant vitamins reflect the oxidative status of the animal, both NG and DM. Clear links between antioxidant vitamins and oxidative status have been demonstrated. Moreover, the changes in antioxidant vitamin levels during Bet supplementation strongly support the importance of betaine.

#### 3.1.3. Comparison of Systemic and Renal Haemodynamics Between NG and DM After Treatment with Bet Alone or Combined with L-NAME

We confirmed here our previous finding that baseline MBP does not significantly differ between NG and DM rats and this was in agreement with our previous finding [40]. As expected, Bet + L-NAME co-treatment increased baseline MBP, both in NG and DM rats. While baseline HR was slightly higher in the non-treated NG than in the DM group, this difference was not visibly altered by Bet pretreatment. However, with L-NAME added, HR decreased in both groups (more pronounced in diabetic rats). Thus, under baseline conditions the tonic influence of *NO* could be important in the regulation of systemic haemodynamics in Bet-treated female rats, independent of the BG status.

Baseline RBF was slightly higher in NG than in DM females. Unexpectedly, after Bet-pretreatment RBF was lower in both NG and DM rats, most probably due to the release of a vasoconstricting factor or deficiency of some vasodilating factor. The latter was likely not *NO* because after L-NAME co-treatment renal perfusion was even lower than after Bet given alone, most probably due to the lack of vasodilating *NO* after chronic NOS inhibition.

We found that baseline RVR was lower in non-pretreated NG compared with DM rats. This difference was abolished after Bet treatment, which unexpectedly resulted in an increase in RVR, significantly so in NG but not in the DM group. The increases were observed after L-NAME in both groups and, again, baseline RVR was distinctly lower in NG than in DM rats.

On the whole, we found that any differences in the baseline vascular conditions between normo- and hyperglycaemic rats can be modulated by Bet treatment, which indicates that the drug exerts renal and extrarenal vasoactive actions irrespective of the current glycaemia.

It should be emphasised, however, that the data from group DM with Bet + L-NAME co-treatment should be interpreted with great caution, due to the small number of experiments analysed. Under this specific co-treatment in diabetic rats, some of them showed high sensitivity to anaesthesia and/or surgery, and they died in the initial phase of the acute experiment. This hypersensitivity confirmed the high importance of endogenously released *NO* in control of body functions, particularly in diabetes.

#### 3.1.4. Comparison of Renal Regional Blood Perfusion Between NG and DM After Treatment with Bet and Bet Combined with L-NAME

Similarly as with RBF, baseline CBF was distinctly higher in NG than in the DM group; however, no such difference was seen in Bet-pretreated rats. Interestingly, in L-NAME-treated groups CBF was, again, higher in NG compared with DM females but the mean values were not distinctly different from baseline values of the respective non-pretreated counterparts. The latter confirms that irrespective of the actual glycaemia level, the *NO* contribution to the regulation of blood supply to the cortex was negligible.

Oxidative stress has been shown to be associated with the onset and progression of DM. We might speculate (no measurement here of tissue ROS level) that lower renal perfusion in DM rats could be due to increased ROS synthesis. As was summarised by Wronka et al. [46], the pathogenetic basis of diabetes and its complications (vascular and neurological) is oxidative stress. Persistent hyperglycaemia promotes the production of free radicals in ß-cells through the mitochondrial respiratory chain [46].

Noteworthily, unlike with cortical perfusion, neither basal outer nor inner medullary perfusion (OM-BF and IM-BF) was affected by diabetes. However, after Bet pretreatment both OM-BF and IM-BF were significantly higher in the DM group. In contrast, in NG rats lower basal OM-BF was shown without post-Bet alteration in IM-BF. Interestingly, after Bet + L-NAME co-treatment in the NG group, baseline OM-BF was higher than that seen after Bet alone; again, no difference was observed in IM-BF. However, in DM rats L-NAME abolished the Bet impact and neither OM-BF nor IM-BF differed from the baseline for Bet- or untreated counterparts. These findings indicate that the issue is complex; as expected, Bet supplementation can improve medullary perfusion in diabetic females, which can depend on intact NOS. On the other hand, exogenous Bet intake in normoglycaemic animals could mediate medullary vasoconstriction. Thus, these results suggest that Bet may affect vascular function in the renal medulla through diverse pathways, depending on the actual glycaemia and/or NOS activity state.

Earlier we reported that in females, neither basal OM-BF nor IM-BF were affected by diabetes or L-NAME (alone) treatment [40]. In the current study, NOS inhibitor was also given in drinking water for four days but the rats received Bet pretreatment. Taken together, data from the current work indicate that Bet can modulate perfusion of the medulla, especially of its outer zone; however, depending on glycaemia Bet could increase or decrease it. Furthermore, chronic NOS inhibition can abolish the Bet-dependent alteration in outer medullary perfusion. Accordingly, it could be speculated that the used dose of NOS inhibitor was not effective in eliminating the local NOS activity in the medulla. Nevertheless, the L-NAME dose applied induced peripheral vasoconstriction (elevated MBP), increased renal vascular resistance and diminished perfusion of the renal cortex.

Noticeably, in our female rats baseline OM-BF and IM-BF were not altered by diabetes or L-NAME co-treatment, whereas many studies have shown high expression and activity of NOS, especially nNOS, in the medullary zone of the kidney [47,48]. In this context, it is recalled that in our studies with male Wistar NG rats renal *NO* bioavailability was found to be much higher in the medullary compared to the cortical tissue [49] and in a later study NOS inhibition tended to decrease medullary perfusion, peripheral vasoconstriction (MBP elevation) and a diminution in RBF [50].

Generally speaking, with higher baseline NOS activity in the medulla than in the cortex in either sex, in females it can be further increased during the initial phase of diabetes [14,51]. It is unclear and perplexing why in our recent and current studies chronic L-NAME treatment caused peripheral and renal cortical vasoconstriction in both female and male SD rats whereas it did not alter the circulation in the medulla [40,52]. Thus, regardless of the sex, perfusion of the renal medulla seems well protected, which can be related to its assumed role in control of BP regulation [41,53].

#### 3.1.5. Comparison of Renal Excretion Between NG and DM After Treatment with Bet Alone and Bet Combined with L-NAME

We found earlier that in conscious female rats renal excretion was much lower in NG than in DM but the difference was abolished by anaesthesia [40]. Here again, basal V, U_Na_V, and U_K_V were slightly but significantly higher in normo- than in hyperglycaemic rats. Since in the former group RBF and CBF tended to be even lower and MBP was comparable, the data suggested higher renal tubular fluid reabsorption in DM rats. This accords with the presumable prevalence of tubular antidiuretic/antinatriuretic over diuretic/natriuretic factors in diabetic rats. Surprisingly, Bet pretreatment did not alter V and U_Na_V in DM rats, whereas it decreased them in the NG group, even though in the latter Bet treatment was accompanied by higher blood pressure and pressure-dependent diuresis and natriuresis could be expected. Simultaneously, potassium excretion was significantly lower after Bet treatment, in both NG and DM rats. However, this was not the case for the excretion of U_osm_V, which did not differ between NG and DM groups, similarly in Bet-treated and untreated animals. Apparently, Bet can improve water, sodium and potassium tubular reabsorption in NG rats whereas in DM rats it does this with potassium only. Thus, the net effect of Bet on renal excretion depends on the actual glycaemia.

The baseline difference in the excretion between NG and DM rats (higher V, U_Na_V and U_K_V in the former) was clearly reversed after Bet treatment combined with NOS blockade (Table 2). This may have been due to a post Bet + L-NAME decrease in renal haemodynamics (RBF and CBF) in the NG group (reduced excretion), hence also the difference in excretion between NG and DM rats. However, we showed that systemic blockade of *NO* synthesis in diabetic rats may result in an increase in renal excretion [40]. Here, L-NAME administration was preceded by Bet pretreatment and the renal excretion effects were smaller than in the former study [40]. Noteworthily, L-NAME co-treatment with Bet was associated with a blood pressure increase irrespective of actual glycaemia; hence, a pressure-dependent renal excretion increase could be expected. On the other hand, under Bet treatment the tubular sodium and water transport might be tonically reduced by *NO* more in DM than in NG rats; there is evidence that *NO* synthesis is enhanced in diabetes, at least at the early stage [54,55]. Likely, given the concomitant differences between groups in renal circulation and probably also in glomerular filtration rate (GFR), the involvement of renal perfusion and tubular transport changes in the alterations of renal excretion is difficult to unravel.

In summary, data from our study indicate that in diabetic females betaine could modulate *NO* action on renal tubular transport.

### 3.2. Theo Effects: Impact of Bet and Bet + L-NAME Pretreatment

In animals subjected to anaesthesia and surgery, there was no apparent basal tonic influence of the Ado system on the total peripheral vascular resistance (TPVR) and this was not modified by Bet pretreatment. As similarly seen in the earlier study with female SD rats, acute nonspecific A1 and A2 receptor inhibition with Theo did not alter MBP in females drinking pure water (non-pretreatment), irrespective of glycaemia [40], and in the current work this was also shown in animals pretreated with Bet.

#### 3.2.1. Theo-Induced Decrease in MBP (with *NO* Blockade)

We reported recently that under impaired *NO* synthesis, acute Theo caused a significant and persistent decrease in MBP, but only in NG females [40]. Here we demonstrated that if chronic *NO* blockade is preceded by Bet-treatment, Theo can also cause a significant and persistent decrease in MBP in DM rats. This indicates that Bet could also modify *NO* participation in MBP control in diabetic females.

Why in the *NO*-deficient Bet-treated hyperglycaemic rats was the overall TPVR sensitive to the blockade of Ado receptors? Any relation of this sensitivity to blockade of P1R-A2 receptors seems unlikely because the impact of P1R-A2 through *NO* stimulation had been eliminated by the *NO* inhibitor given before Theo administration. However, the impact of *NO*-mediated P1R-A2 action must still be considered: it cannot be excluded that the reason for the MBP decrease was a diminution in cardiac output. In rats adenosine may improve coronary perfusion through an increase in the P1R-A2 activity, even in the absence of *NO*. It was shown that P1R-A2 can induce *NO*-independent dilation of the coronary arteries [56,57,58]. Possibly, inhibition of these receptors by Theo causes coronary vasoconstriction, leading to reduced cardiac output and a decrease in MBP. However, the post-Theo HR increase observed in all experimental groups, regardless of actual glycaemia, argues against this hypothesis.

The reason for the hypotensive response to Theo seen in NG and DM rats with *NO* deficiency could be different basal TPVR because the baseline post-L-NAME level of BP in Bet-treated animals was higher, in both NG and DM groups (this was also the case with baseline RVR values, see Table 2). Possibly, the reason could be higher basal density of vasoconstrictor P1R-A1 in NG and also in DM rats [58].

Our results do not agree with the evidence on Ado-induced relaxation (rather than contraction) described recently in experiments using endothelium-denuded rat aorta isolated from nondiabetic males [59]; however, the results of ex vivo *versus* whole animal studies should be compared with caution.

In our recent study, in addition to the post-Theo blood pressure reduction, *NO*-deficient female NG rats also showed reduced renal inner medullary perfusion (IM-BF [40]. However, here we did not see a parallelism of post-Theo changes in MBP and IM-BF (IM-BF only tended to fall with MBP drop, irrespective of the glycaemia). Thus, our results do not accord with the widespread view on impairment of IM-BF autoregulation in the face of blood pressure alterations [53,60].

#### 3.2.2. Renal Blood Perfusion and Ado Receptor Blockade

Theo administration transiently increased whole kidney perfusion (RBF), similarly in non-pretreated (neither Bet nor Bet + L-NAME) normo- and hyperglycaemic female rats. Dissimilarly, under conditions of Bet treatment alone or combined with *NO* synthase inhibition, Ado receptor blockade induced renal vasodilatation and sustained increased perfusion, also visible in the post-Theo recovery period. The effect was probably due to the abolishment of baseline (pre-Theo) vasoconstriction. However, this effect was seen only in NG rats, whereas in diabetic rats Bet pretreatment did not alter the vascular effect of Theo. Remarkably, in the DM group the slight decrease in RVR (after Bet pretreatment) was similar to the situation with *NO* blockade, when Ado receptor blockade also tended to decrease RVR.

Noteworthily, in Bet-pretreated NG females no significant alterations in OM-BF or IM-BF were seen after Ado receptor blockade, whereas in the DM group a moderate IM-BF decrease was seen. The post-Theo decrease in perfusion could be the effect of elimination of P1R-A2-mediated vasodilation, which seems to be less important in the control of medullary perfusion in NG females. Taken together, our data indicate that Bet can modulate the P1R contribution to control of medullary perfusion, especially of the inner zone, and the action depends on actual glycaemia.

On the whole, under conditions of *NO* deficiency, in response to Ado receptor blockade whole renal perfusion increased moderately; the change was greater in NG than in DM rats. This speaks to a greater vasoconstrictor influence of the Ado system in non-diabetic females (Figure 1). Our results from Ado inhibition experiments accord well with those obtained with application of exogenous Ado. In normoglycaemic males, the renal vasculature was found to be more sensitive to adenosine-mediated vasoconstriction when *NO* synthases were inhibited [61]; we did show such dependency in females when *NO* blockade was preceded by Bet-treatment. Furthermore, in an earlier study with streptozotocin-diabetic rats, the kidney was also clearly more responsive to the vasoconstrictor action of Ado in *NO*-deficient animals [62]; however, this was not observed in our study with females.

Under conditions of *NO* deficiency, there was no difference between NG and DM rats in the post-Theo response of renal outer or inner medulla circulation. However, unlike the increase seen in RBF and CBF, both OM-BF and IM-BF tended to decrease. Possibly, this difference was due to the abolishment of baseline (pre-Theo) vasodilation specific to the medullary vasculature pretreated with Bet; the effect seems to be independent of glycaemia status.

#### 3.2.3. Renal Excretion and Ado Receptor Blockade

Acute intravenous delivery of a non-selective purinergic receptor antagonist (Theo) altered the diuresis but significantly so only in NG female rats, and the difference between NG and DM groups observed before Theo infusion disappeared. Notably, Bet-treatment abolished Theo effects in NG rats, and the difference between NG and DM was also not seen. Different post-Theo effects were observed for the natriuresis which tended to increase visibly more in NG than in DM rats. Without Bet, the difference between NG and DM groups seen before Theo infusion persisted but it did not reach the significance level after Bet pretreatment. On the other hand, Theo significantly increased potassium excretion both in NG and DM females, and the U_K_V level difference between the NG and DM groups remained significant, as was also observed during the basal period. However, after pretreatment with Bet post-Theo U_K_V increased significantly only in diabetic females.

Remarkably, quite different Theo effects were seen for total solute excretion: the post-Theo increase was similar in NG and DM rats, both without and with Bet treatment. Taken together, the data suggest that the tubular action of purinergic receptors in female rats can be modified by Bet; however, the final effect depends on the glycaemia level.

Notably, Theo effects on renal excretion after NOS inhibition differed from those seen in the females treated with Bet alone. Similar directions of the post-Theo effects were shown for diuresis, sodium, potassium, and total solute excretion; however, an increase significantly greater than that shown with intact *NO* synthesis was found in NG rats only. This was unexpected because a concurrent decrease in MBP would rather provoke a decrease in renal excretion. Again, this suggests that the excretion increases after Ado receptor blockade were not simply the consequence of systemic haemodynamics changes but might also be related to decreasing tubular reabsorption. Taken together, this suggests that *NO*’s influence on the tubular action of purinergic receptors in female rats is dependent on glycaemia.

#### 3.2.4. Tissue *NO* and Ado Receptor Blockade

Although Theo did not induce significant tissue *NO* changes in any experimental group, some between-group differences were apparent. In non-pretreated animals, Theo altered tissue *NO* signal to a level somewhat below or above baseline in NG or DM females, respectively (Figure 1). Such an effect could be expected in NG rats because inhibition of P1R-A2 by Theo can cause a decrease rather than an increase in the kidney tissue availability of *NO* [63]. After Bet pretreatment, the difference in Theo’s effect on tissue *NO* was greater between NG and DM females and became significant. However, the renal NO signal changes were reversed: Theo increased the *NO* signal in NG and decreased it in DM rats. Interestingly, these alterations were parallel to changes in IM-BF. No such discrepancy between groups was shown for other renal blood perfusion parameters (RBF, CBF or OM-BF). This indicates that Bet can modify the impact of P1 receptors, particularly on medullary perfusion and *NO* availability.

Notably, under conditions of inhibited *NO* synthesis, acute antagonism of purinergic receptors induced inverse changes in tissue *NO:* a decrease in NG and an increase in DM females. This was unexpected because in both groups a parallel decrease in MBP and IM-BF was seen; one should here consider stimulation of *NO* release from a source other than NOS, e.g., from S-nitrosothiols, the main form of *NO* storage within the vasculature [64].

Interestingly, in males unidirectional changes in blood pressure and medullary perfusion were associated with an increase of tissue *NO* in the DM group while in the NG group Theo did not alter MBP, IM-BF or tissue *NO* [52].

Taken together, a potential mechanism for *NO* release in medullary tissue could be less important in normo- than in hyperglycaemia. However, a comparison of the current data with that from the recent study in males [52] indicates that the ultimate effect of Ado receptor blockade on tissue *NO* availability could differ between sexes.

## 4. Materials and Methods

### 4.1. Experimental Animals

The study protocols were accepted by the Ethical Committee for the use of experimental animals (II Local Ethical Committee, Warsaw, Poland), permission no. WAW2/045/2022, and were in agreement with the National Institutes of Health Guide for the Care and Use of Laboratory Animals and the European Union Directive 63/2010. We used out-bred SD female rats (Tac:SD), obtained from the intramural animal breeding house (total number, *n* = 62), non-diabetic (NG) or with induced diabetes (DM). They were aged 6–7 weeks before hyperglycaemia was induced (see below). The animals were housed in groups of 2–4, under a 12:12 h light–dark cycle, and had free access to tap water and standard rat chow (dry pellets with 0.25% Na *w*/*w*, SSNIFF GmbH, Soest, Germany). Rats were kept in stable groups, both during breeding and before the primary chronic experiment, which ensured that the oestrus cycle was synchronised. Additionally, at the beginning of the experiment, a vaginal smear was performed to determine the phase of the cycle.

### 4.2. Studies with Conscious Animals

Just before the start of chronic observations, the animals were randomly divided into two groups. In one, STZ was given to induce hyperglycaemia, whereas in the other its solvent was given to keep the animal in the NG state. Each time, four rats were injected (two per STZ and two per solvent), to have at the same time both NG and DM in the same conditions.

#### 4.2.1. Experimental Diabetes Induction

**Figure 3:**
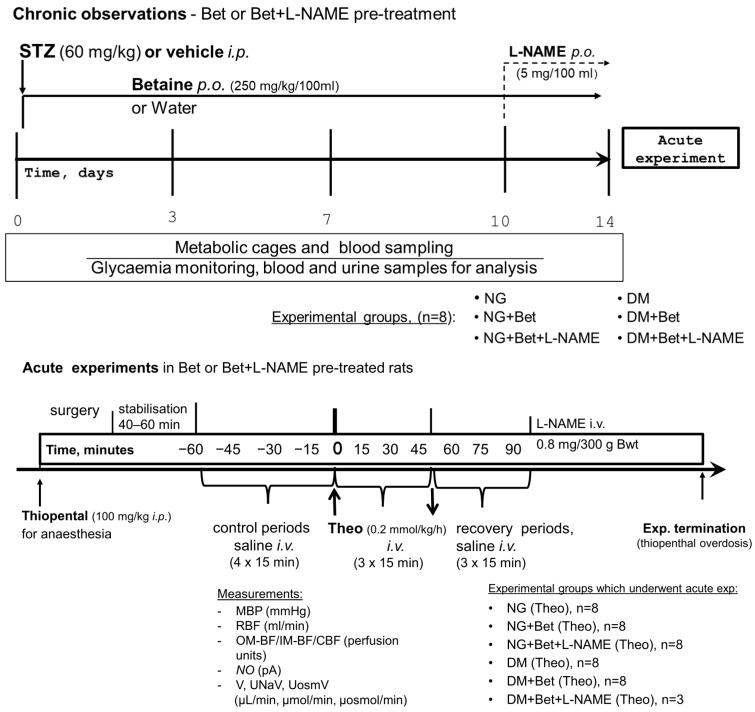
Diagram of the study. (**Upper panel**) Chronic part of the study. One week before the rats reached 7 weeks of age, they were accustomed to experimental conditions and thereafter randomly assigned to the experimental protocol: with STZ to induce diabetes (DM) or vehicle (NG- normoglycaemic), and pretreated with Bet (betaine alone) or Bet + L-NAME (co-treated with L-NAME) given in drinking water. Blood sample collection and 24 h metabolic cage observations and urine sampling conducted before (day 0) and 3, 7, 10, 14 days after STZ or solvent injections are marked. (**Lower panel**) Acute part of the study—anaesthetised rats were surgically prepared for kidney function measurements before (control periods) and during and after (recovery) Theo infusion. Abbreviations: STZ—streptozotocin; L-NAME (a non-selective nitric oxide synthase inhibitor); Theo—theophylline, a non-selective P1 receptors antagonist; MBP—mean blood pressure; RBF, OM-BF, IM-BF, CBF—renal, outer, inner medullary and cortical blood flow, respectively; *NO*—tissue nitric oxide; V, U_Na_V, U_osm_V—urine, sodium and total solutes excretion, respectively.

STZ 60 mg/kg (Santa Cruz Biotechnology, Inc., Dallas, TX, USA), prepared in a solution of citrate buffer (0.05 mol/L, pH 4.5) directly before injection, was given as a single intraperitoneal (*i.p.*) injection to induce hyperglycaemia. Starting from the day before STZ injection until the acute experimental rats’ BWt and BG were checked. On days 3, 7, and 10 or 14 after STZ was given, animals were fasted for two hours before BG level was determined (Figure 3, upper panel). The females with BG exceeding 300 mg/dL measured 72 h after STZ injection and persisting so increased until the end of the observation were considered diabetic.

#### 4.2.2. Betaine Administration

Starting from the day before STZ injection until the acute experiment, in randomly selected NG and DM animals, Bet (Sigma, Poznań, Poland) was given orally for fourteen days (Bet_14_) by admixing the Bet powder, 250 mg/kg/100 mL, in drinking water (Figure 3, upper panel). Each time, one person prepared each solution in bottles with coded markings (to maintain the blinding condition). Thereafter, another person put bottles into cages. The concentration of the drug was adjusted daily on the basis of body weight (BWt) and water consumption on the previous day.

#### 4.2.3. L-NAME Administration

Before the acute experiment, in a random manner in NG and DM animals, L-NAME, a non-selective NOS inhibitor (N(G)-nitro-L-arginine methyl ester; Sigma, Poznań, Poland), was administered orally for four days (L-NAME_4_) by adding the powdered substance, 5 mg/100 mL, to drinking water (Figure 3, upper panel). The exact handling of solution in bottles was as described for betaine (see above).

The rationale to use the same L-NAME concentration for NG and DM rats, even though the latter drank more water, was that in DM rats the production of renal *NO* was often found to be elevated [65]. The similar effectiveness of the higher L-NAME dose in DM animals was confirmed by measuring the response of tissue *NO* signal to a post-experiment *i.v.* injection of L-NAME bolus (see the details below).

In our previous studies, an analogous approach resulted in effective inhibition of endogenous *NO* production in rats on standard and high sodium intake; the latter showed higher water intake but also produced more *NO* [50].

### 4.3. Studies with Anaesthetised Animals

#### 4.3.1. Surgical Preparation for the Experiment

For anaesthesia, a single *i.p.* injection of thiopental solution was given (Figure 3, lower panel). Sodium thiopental (100 mg/kg, Thipen, Samarth Life Science, Tumkur, India) provided stable anaesthesia, at least for the next four hours. The required depth of anaesthesia was verified by control of HR and repeated control of pedal and corneal reflexes. To maintain stable body temperature at about 37 °C (measured in rectum) during surgery and experiment, the animals were placed on a surgery table with servo-controlled heating. To ensure free airways, a polyethylene tube was placed in the trachea, whereas for fluid infusions and MBP, and HR measurements (Stoelting transducers and blood pressure meter, Wood Dale, IL, USA), the jugular vein and the carotid artery were cannulated, respectively. To preserve plasma volume, 3% bovine serum albumin solution was infused by intraoperative *i.v.* at 3 mL/h. A subcostal flank incision allowed exposure of the left kidney, which was fixed in a plastic holder. To collect the urine, a thin plastic tube was inserted into the ureter, and the volume of urine was measured gravimetrically. The details of measurements of RBF, using a non-cannulating ultrasound probe, as well as the laser-Doppler technique to determine the renal regional flows through the cortex (CBF), outer and inner medulla (OM-BF and IM-BF, respectively) flows were described in detail previously [50,52].

For measurement of tissue *NO* changes in the kidney medulla, we performed amperometric measurement of *NO* signal, utilised in our laboratory for several years, using a needle-shaped ISO-NOP 200 sensor (0.2 mm in diameter), connected with a Free Radical Analyser containing *NO* meter (TBR 4100, World Precision Instruments, Inc., Sarasota, FL, USA). The probe was inserted into the medulla to the depth of 5–7 mm determined vertically from the kidney surface. *NO* measurement details and calibration of the results were described previously [49,50,52] and placed here in Supplementary File: S1 Materials and methods, Subsection S1.1. Calibration of *NO* probe.

#### 4.3.2. Experimental Procedures

In each experimental group, after completing the preparation for the experiment followed by the recovery period (45–60 min), four control periods (15 min each) were made to establish the baseline of renal excretion and haemodynamics. Then, during three 15 min experimental periods, Theo at the dose of 0.2 mmol/kg/h or its solvent (0.9% of sodium chloride) was given intravenously with subsequent periods of recovery (Figure 3, lower panel). These experimental steps were used in six groups of rats (*n* = 8, each) described below: (1) NG rats, non-pretreated (acute Theo infusion)—NG (Theo); (2) NG rats, Betaine_14_-pretreated (acute Theo infusion)—NG + Bet_14_ (Theo); (3) NG rats, Betaine_14_ and L-NAME_4_-pretreated (acute Theo infusion)—NG + Bet + L-NAME (Theo); (4) DM rats (acute Theo infusion)—DM (Theo); (5) DM rats, Betaine_14_-pretreated (acute Theo infusion)—DM + Bet_14_ (Theo); (6) DM rats, Betaine_14_ and L-NAME_4_-pretreated (acute Theo infusion)—DM + Bet + L-NAME (Theo).

Additional groups were formed for plasma and tissue vitamin level measurements in non-pretreated and Bet-pretreated rats:

NG, *n* = 3, DM, *n* = 3, NG + Bet_14_, *n* = 4, DM + Bet_14_, *n* = 4. (1)

### 4.4. Analytical Procedures and Calculations

The level of BG was determined using an ACCU-CHECK Active Model GC glucometer (Roche, Mannheim, Germany). The volume of urine was measured gravimetrically. To measure the osmolality of urine (U_osm_), a cryoscopic osmometer Osmomat 030 (Gonotec, Berlin, Germany) was used. The plasma/urine concentration of sodium (P_Na_/U_Na_) and potassium (P_K_/U_K_) were determined using a flame photometer (Flame Photometers, BWB Technologies, Newbury, UKU_K_). The renal excretion parameters of timed V, U_osm_V, U_Na_V and U_K_V excretion were determined using the common formulas and standardised to g kidney weight (U_X_V/g KW).

It should be mentioned that plasma and urine samples were collected and stored in tubes with coded markings until the analytical procedures were completed.

#### Vitamin Measurements

Whole blood was collected from a cannulated rat. Immediately after separating, the kidneys and liver were removed and directly washed out using ice-cold 0.9% NaCl solution and dried. Thereafter, the wet weights were determined, and the organs were stored at −20 °C until the planned analysis. Determination of retinol (Vit. A) and α-tocopherol (Vit. E) in rat serum was conducted by the isotope dilution method using HPLC coupled with LC-MS/MS (high-performance liquid chromatography and tandem mass spectrometry, respectively). Prior to chromatography, the tissue was freeze-dried. (Vitamin measurement details were described in Supplementary File: S1 Materials and methods, Subsection 1.2. Vitamin measurements).

### 4.5. Statistical Methods

Values were presented as the mean ± standard error of the mean (SEM) as a measure of data dispersion, with statistical significance defined as *p* < 0.05. To define the n number needed to arrive at reliable determination of significant differences, appropriate power analysis was first performed. It was based on the statistics dedicated to the ethics approval. The optimal number of animals in a group was calculated by taking blood glucose as one of the observed parameters during treatment with the test substance, using the formula: n ≥ [(tγ + t1-α/2)^2^ • s2]/(μ1 − μ0)^2^. A power analysis was performed with the following assumptions: mean blood glucose level in untreated rats (established in previous experiments) on the day of test initiation μ0 = 7 mmol/L; expected mean (obtained in previous experiments) change in blood glucose after treatment (STZ injection) μ1 = 24 mmol/L; variance (obtained in previous experiments) s = 17.9; assumed power of the test (80%); tγ for the standard normal distribution = 0.68; t1-α/2 with the assumed error α = 0.05 for the standard normal distribution is 1.96; n ≥ [(0.68 + 1.96)^2^ • 17.92]/(22.2 − 7.2)^2^; *n* ≥ 7.7. If two data sets within one group or two groups were considered for comparison, a two-tailed Student’s *t*-test was used (paired or unpaired samples, respectively). In case of multiple comparisons (within each group), data were analysed using repeated-measurement analysis of variance (ANOVA) followed by Bonferroni’s test. Comparisons between the treatments were first analysed by repeated-measures multivariate ANOVA, followed by Tukey’s post hoc test. Statistical analyses were performed using version 10.0. of STATISTICA software (StatSoft Polska Sp. z o.o., Kraków, Poland).

## 5. Conclusions

In the present study, we showed that the dissimilarity in the baseline vascular state between NG and DM rats can be modified by dietary Bet supplementation, which indicates that this compound is vasoactive in and outside the kidney, irrespective of the current glycaemia. However, under normoglycaemia, dietary Bet modified blood flow only in the outer medulla, whereas under diabetic conditions both outer and inner medullary perfusion was altered. Renal excretion appears to be modified by dietary Bet treatment depending on glycaemia status; it increases water, sodium, and potassium tubular transport in normoglycaemia or only potassium reabsorption in hyperglycaemia.

When dietary Bet was combined with blockade of *NO* synthesis, it appeared that the tonic influence of *NO* was important in the regulation of systemic but not whole kidney haemodynamics. However, under hyperglycaemia, *NO* availability was important for Bet-induced vasodilatation in the renal medulla. Moreover, in diabetic females, Bet could modulate *NO* action on renal tubular transport.

Our study, applying a blockade of Ado and *NO* superimposed on dietary Bet supplementation, sheds new light on the functional association of the two systems in NG and DM animals. We showed that in Bet pretreated female rats:

(i) With intact *NO* synthesis, a tonic vasoconstrictor influence of the Ado system on the resistance of the peripheral vessels was seen. However, within the kidney vasculature, the vasoactive influence of the Ado system was not altered by hyperglycaemia and not modified by Bet pretreatment.
(ii) Under *NO* deficiency, Ado receptor blockade induced an MBP decrease of unclear origin. This was in contrast to the status without Bet-treatment, when arterial pressure lowering was seen only in NG rats. However, renal vasodilatation, seen in NG but not in DM rats, suggested that the vasoconstrictor influence of the Ado system in the female sex could depend on the actual glycaemia level.
(iii) Another fresh insight was that in the renal medulla (especially in the inner zone), the role of P1R (Ado-dependent receptors) on blood supply may depend on the actual glycaemia, whereas the P1R impact on tissue *NO* availability may be related to both glycaemia and the activity status of *NO* synthases.
(iv) Irrespective of the status of *NO* synthesis (intact or deficient), the final tubular action of P1R on water and solute transport depended on the actual glycaemia.

On the whole, here we have provided information on the complex role of betaine in modification of the effects of already well-explored systems (*NO* and adenosine) on renal function in experimental diabetes in female rats. Such basic knowledge seems prerequisite for future clinically-oriented research.

## Figures and Tables

**Table 1:** Body weight, blood and plasma (tail vein samples, upper section) and the data of 24 h observations in metabolic cages (lower section) for the samples collected before (day 0) and 10 or 14 days after STZ or solvent injections in female Sprague–Dawley (Tac:SD) rats, NG or DM (normoglycaemic or diabetic, respectively): left side—rats pretreated for 14 days with betaine; right side—rats pretreated for 14 days with betaine and co-treated (+) for the last 4 days with L-NAME (values shown in bold).

Parameter		Days After Buffer or STZ Injection Betaine Pretreatment			Days After Buffer or STZ Injection Betaine + L-NAME Pretreatment		
		0	10	14	0	10	14
Body weight (g)	NG	246 ± 2	249 ± 3	249 ± 3	253 ± 4	254 ± 9	**249 ± 7**
	DM	245 ± 2	217 ± 9 *#	210 ± 11 *#	262 ± 8	221 ± 6 *#	**210 ± 7** *#
Glycaemia (mg/dL)	NG	194 ± 10	191 ± 20	182 ± 11	189 ± 13	155 ± 5 *	**145 ± 6** *
	DM	186 ± 8	540 ± 24 *#	562 ± 22 *#	155 ± 6	485 ± 30 *#	**515 ± 20** *#
Haematocrit (%)	NG	45 ± 1	43 ± 1	45 ± 1	45 ± 1	44 ± 1	**46 ± 2**
	DM	43 ± 0	45 ± 1	45 ± 1 *	45 ± 1	46 ± 1	**46 ± 1**
Water intake (mL/24 h)	NG	26 ± 1	22 ± 2	22 ± 1	24 ± 2	23 ± 3	**17 ± 2**
	DM	26 ± 4 #	95 ± 4 *#	102 ± 11 *#	28 ± 3	76 ± 7 *	**77 ± 6** *
Urine flow (mL/24 h)	NG	10 ± 1	7 ± 1 *	8 ± 2	11 ± 1	6 ± 1 *	**6 ± 1** *
	DM	10 ± 1	71 ± 7 *#	77 ± 10 *#	14 ± 2	68 ± 7 *	**68 ± 5** *
Urine osmolality (mosmol/kg H_2_O)	NG	1600 ± 95	2120 ± 190 *	2065 ± 195 *	1520 ± 105	1890 ± 215	**2005 ± 120** *
	DM	1845 ± 145	1185 ± 60 *#	1135 ± 75 *#	1515 ± 70	1295 ± 115	**1360 ± 150**
Total solute excretion (mosmol/24 h)	NG	15 ± 1	15 ± 1	16 ± 2	17 ± 1	11 ± 2	**13 ± 3**
	DM	13 ± 2	80 ± 8 *#	87 ± 11 *#	20 ± 1	80 ± 8 *#	**82 ± 5** *#
Urine sodium excretion (mmol/24 h)	NG	1.2 ± 0.1	1.0 ± 0.1	1.3 ± 0.2	1.0 ± 0.1	2.1 ± 1.3	**0.9 ± 0.2**
	DM	1.0 ± 0.2	1.7 ± 0.3 *	2.1 ± 0.3 *	1.5 ± 0.1	1.8 ± 0.2	**1.7 ± 0.1**

The values are means ± SEM; *n* = 8. STZ—streptozotocin; L-NAME (NG-nitro-L-arginine methyl ester, a non-selective nitric oxide synthase inhibitor) and betaine were dissolved in drinking water. * significantly different from day 0, # significantly different from NG.

**Table 2:** MBP (mean blood pressure), HR (heart rate), renal haemodynamics and excretion in NG (normoglycaemic) or DM (hyperglycaemic) female rats, non-pretreated or pretreated with Bet (betaine) and Bet + L-NAME (Bet pretreatment and co-treatment with L-NAME for the last 4 days).

Parameter	Pretreatment	NG	DM
MBP	None	124 ± 6	124 ± 3
(mmHg)	Bet	133 ± 1	121 ± 4
	Bet + L-NAME	145 ± 2 *#	137 ± 3 *#
HR	None	345 ± 15	303 ± 10 †
(beats/min)	Bet	350 ± 2	300 ± 5 †
	Bet + L-NAME	320 ± 10 #	254 ± 11 *†#
RBF	None	5.1 ± 0.3	3.8 ± 0.3 †
(ml/min/g of kidney weight)	Bet	3.9 ± 0.3 *	3.1 ± 0.1 *†
	Bet + L-NAME	3.0 ± 0.3 *#	1.9 ± 0.3 *#
RVR	None	25 ± 3	35 ± 2 †
(mmHg min/mL)	Bet	40 ± 4 *	40 ± 2
	Bet + L-NAME	54 ± 5 *#	86 ± 15 *†#
CBF	None	710 ± 20	520 ± 25 †
(perfusion units)	Bet	570 ± 45 *	550 ± 40
	Bet + L-NAME	630 ± 30	515 ± 15 †
OM-BF	None	210 ± 25	150 ± 20
(perfusion units)	Bet	140 ± 10 *	415 ± 30 *†
	Bet + L-NAME	265 ± 30 #	250 ± 70
IM-BF	None	205 ± 45	155 ± 15
(perfusion units)	Bet	240 ± 45	220 ± 30
	Bet + L-NAME	220 ± 25	245 ± 50
V	None	8.2 ± 1.4	6.5 ± 0.6 †
(µL/min/g of kidney weight)	Bet	5.1 ± 1.0 *	5.6 ± 1.1
	Bet + L-NAME	4.6 ± 0.6 *	8.8 ± 2.6
U_osm_V	None	5.9 ± 0.6	6.7 ± 0.7
(µosmol/min/g of kidney weight)	Bet	4.2 ± 0.9	5.4 ± 1.3 †
	Bet + L-NAME	4.1 ± 0.4	9.1 ± 3.1
U_Na_V	None	0.9 ± 0.2	0.5 ± 0.1 †
(µmol/min/g of kidney weight)	Bet	0.5 ± 0.1 *	0.4 ± 0.1
	Bet + L-NAME	0.3 ± 0.1 *	0.9 ± 0.3
U_K_V	None	0.9 ± 0.1	0.4 ± 0.1 †#
(µmol/min/g of kidney weight)	Bet	0.4 ± 0.1 *	0.1 ± 0.1 *
	Bet + L-NAME	0.6 ± 0.1 *	0.7 ± 0.1 #

L-NAME (NG-nitro-L-arginine methyl ester, a non-selective nitric oxide synthase inhibitor); RBF—whole kidney blood flow; RVR—renal vascular resistance; CBF, OM-BF, IM-BF—cortical, outer and inner medullary blood flow (laser-Doppler flux), respectively. V—urine flow; U_osm_V, U_Na_V, U_K_V—the excretion of total solutes, sodium and potassium, respectively. The values are means ± SEM; *n* = 6–8, each group with exception for DM Bet + L-NAME, *n* = 3; * significantly different from the corresponding non-pretreated group, †—significantly different from the NG group, #—significantly different from the corresponding Bet pretreated group.

**Table 3:** Tissue *NO* (nitric oxide) in NG (normoglycaemic) or DM (hyperglycaemic) female rats, non-pretreated or pretreated with Bet (betaine alone) or Bet + L-NAME (Bet combined with L-NAME).

Experimental Group	Tissue *NO* [nA]	Experimental Group	Tissue *NO* [nA]
NG non-pretreated	270 ± 33	DM non-pretreated	260 ± 15
NG + Bet	245 ± 40	DM + Bet	350 ± 45 *
NG + Bet + L-NAME	333 ± 42 #	DM + Bet + L-NAME	294 ± 21

The values are means ± SEM for the last two urine collection control periods (U3 and U4); *n* = 6–8, each group with an exception for DM Bet + L-NAME, *n* = 3; L-NAME—NG-nitro-L-arginine methyl ester non-selective *NO* synthase inhibitor; * significantly different from the corresponding untreated group; # significantly different from the corresponding Bet treated group.

## Data Availability

Data is contained within the article and Supplementary Materials. Raw data are available from the corresponding author upon reasonable request.

## Supplementary Materials

The following supporting information can be downloaded at: https://www.mdpi.com/article/10.3390/ijms27094076/s1. Ref. [66] is cited in Supplementary Materials.

## Abbreviations

The following abbreviations are used in this manuscript:

A1R	Purine receptor P1 subtype A1
A2R	Purine receptor P1 subtype A2
Ado	Adenosine
Bet	Betaine
Bwt	Body weight
BG	Blood glucose
CBF	Cortical blood flow
CVD	Cardiovascular disease
DM	Diabetes, *diabetes mellitus*
ED	Endothelial nitric oxide synthase
H_2_O_2_	Hydrogen peroxide
Hct	Haematocrit
HR	Heart rate
HT	Hypertension
*i.p.*	Intraperitoneal/y
*i.v.*	Intravenous/y
IM-BF	Inner medullary blood flow
L-NAME	NG-nitro-L-arginine methyl ester, a non-selective nitric oxide synthase inhibitor
MBF	Medullary perfusion
MBP	Mean blood pressure
NADPH	Nicotinamide adenine dinucleotide phosphate
NG	Normoglycemia
nNOS	Neuronal nitric oxide synthase
*NO*	Nitric oxide
NOS	Nitric oxide synthase
NOSI	Nitric oxide synthase inhibitor
OM-BF	Outer medullary blood flow
OS	Oxidative stress
P1-A1	Purine receptor P1 subtype A1
P1-A2	Purine receptor P1 subtype A2
P1R	Purine receptor type P1
RBF	Renal blood flow
ROS	Reactive oxygen species
RVR	Renal vascular resistance
STZ	Streptozotocin, diabetes
Theo	Theophylline, a non-selective P1R antagonist
TPVR	Total peripheral vascular resistance
U_K_V	Renal potassium excretion
U_Na_V	Renal sodium excretion
U_osm_V	Renal total solute excretion
V	Urine flow, diuresis
Vit. A	Vitamin A
Vit. E	Vitamin E
