# Supplementary material for: Betaine Alters the Interplay of the Adenosine and NO Systems in the Control of Renal Regional Haemodynamics and Excretion in Diabetic Female Rats
Source: Int J Mol Sci. 2026 May 2;27(9):4076. doi: 10.3390/ijms27094076 (PMC13163498; doi:10.3390/ijms27094076)
Supplement: Supplementary file 1 [file ijms-27-04076-s001.zip › Supplementary file S 1 Materials and methods _15-01-2026 .pdf]

Supplementary Materials:

File S1 Materials and methods -

Subsection 1.1. - Calibration of *NO* probe

Subsection 1.2. - Vitamin measurements

---------------------------------------------------------------------------------------------------------------

1.1. - Calibration of *NO* probe.

For measurement of tissue *NO* signal in the kidney tissue, a needle-shaped ISO-NOP 200 sensor (0.2 mm in diameter), connected with Nitric Oxide Meter (TBR 4100, World Precision Instruments, Inc., Sarasota, FL, USA), was inserted vertically into the medulla, to the depth of 5–7 mm from the kidney surface. Minimal *NO* current measured after L-NAME dose (2.4 mg/kg *i.v.*) given at the end of experiments (after recovery period) was taken as tissue *NO* signal zero level [44]. At the end of experiments the positioning of the laser-Doppler and *NO* probes was examined at the kidney's cross-section.

To verify *in vitro* responsiveness of the *NO* sensor, a curve relating the readings (nA, nanoampers) to known increasing concentrations of *NO* released from S-nitroso-N-acetyl-d,l penicillamine (SNAP) was established as recommended by the manufacturer of the equipment and described by Zhang and Broderick [S1]. This allows to express the results of *in vivo* studies in nA, if needed.

In a previous *in vivo* study the tests confirmed that intravenous administration of L-NAME (NO synthases inhibitor) decreased tissue *NO* signal in a dose-dependent manner whereas infusion of SNAP (*NO* donor) directly into renal artery increased it [44]. Notably, the changes induced by intravenous L-NAME, both in *NO* signal and haemodynamics, were strongly reduced in animals pretreated with oral L-NAME [45]; the same was observed in the present study. Moreover, we found here that an *i.v.* bolus of L-NAME, given after completing of acute experiments without Theo infusion, in NG and DM rats chronically pretreated with L-NAME (in fact with different dose of L-NAME), induced only a minimal 1-2% decrease in tissue *NO* signal, quite similar in NG and DM groups. This indicates that the chronic dosage was adequate and maximally efficient in both diabetic and normoglycaemic animals.

References: [S1] Zhang X, Broderick M. Amperometric detection of nitric oxide. Mod Asp Immunobiol. 2000; 1: 160-165.

1.2. - Vitamin measurements

*Chemicals and reagents*The commercial retinol standard, supplied by Supelco (Germany), was purchased as an ethanolic solution with a concentration of 100 µg/mL and containing 1% butylated hydroxytoluene (BHT). The corresponding isotopically labelled internal standard, purchased in the form of [$^2$H5]-retinyl acetate, together with the deprotection solution, was purchased from Alsachim (France). The commercial $\alpha$-tocopherol standard and its isotopically labelled analogue were obtained from TRC (Canada). BHT was purchased from Sigma-Aldrich (Germany). The rest of the chemicals and solvents used for sample preparation and analysis were of LC-MS/MS grade and purchased from VWR (USA). Certified reference materials in the form of lyophilized human serum, used as quality controls, were supplied by Chromsystems (Germany). Mass Spect Gold Human Serum with Ultra-Low Vitamin E and A was purchased form Golden West Diagnostics (Temecula, USA).

*Sample collection*

To obtain serum samples, whole blood was collected from a cannulated rat into the clot activator. The kidneys and liver were harvested immediately after sectioning and directly rinsed in ice-cold physiological saline solution (0.9% NaCl). Then, the wet weights were recorded, and the samples were

stored at -20°C until the planned analysis. Prior to LC–MS/MS analysis, the tissues were subjected to freeze-drying.

*Sample preparation*

Before serum sample preparation, they were thawed and allowed to reach room temperature. Then, a volume of 100 µL of the serum was transferred into a 1.5 mL Eppendorf tube. A volume of 200 µL of internal standards solution in ethanol, with the addition of butylated hydroxytoluene (BHT) (1%) as a stabilizing agent, was added and the mixture was vortexed (4200 rpm, 30 s) to precipitate proteins. Sample preparation was then carried out using liquid–liquid extraction (LLE) with a hexane containing BHT at a concentration of 1 mg/mL. For this purpose, 500 µL of the extracting solvent was added to the mixture, followed by vortexing (4,200 rpm, 5 s) and shaking (2000 rpm, 5 min). The obtained mixture was centrifuged (14,000 rpm, 6 min) to ensure efficient phase separation. A volume of 400 µL of the upper organic phase (hexane layer) was transferred into a new 1.5 mL Eppendorf tube. The extraction procedure was repeated twice by adding an additional 500 µL of the extracting solvent and following the steps of the described above. After centrifugation, 450 µL of the upper organic phase (hexane layer) was collected and combined with the hexane fraction obtained in the first extraction. The combined layers were then evaporated to dryness under a stream of nitrogen at 30°C, and the dry residue was reconstituted in 200 µL of isopropanol (IPA) containing 1% BHT. Subsequently, 150 µL of the sample was transferred into a round-bottom 96-well polypropylene plate and analyzed.

The lyophilized tissue samples (liver and kidney) were stored at –20°C and brought to room temperature prior to preparation. For homogenization, tissues were ground in a ceramic mortar before further preparation. Then a portion of 20 mg of homogenized tissue (for vitamin E determination in liver and kidneys and for vitamin A determination in kidneys) or 5 mg (for vitamin A determination in liver) was weighed into a 1.5 mL Eppendorf tube. Then, 500 µL of an internal standard solution in acetonitrile containing 1% BHT was added, after which the samples were shaken at 1100 rpm for 20 minutes and then centrifuged at 14,000 rpm for 6 minutes. A 400 µL volume of the supernatant was transferred into a fresh 1.5 mL Eppendorf tube, and an LLE procedure was carried out using hexane containing BHT at a concentration of 1 mg/mL. For this purpose, 750 µL of the extracting solvent was added to the supernatant, the mixture was vortexed (4200 rpm, 5s) and then shaken (1100 rpm, 7 min). The mixture was then centrifuged (14,000 rpm, 6 min) to ensure complete phase separation. A volume of 600 µL of the upper organic phase (hexane layer) was transferred into a new 1.5 mL Eppendorf tube. The extraction procedure was repeated twice by adding an additional 750 µL of the extracting solvent and following the steps of the described protocol. After the second centrifugation, 700 µL of the upper organic phase (hexane layer) was collected and combined with the hexane fraction obtained in the first extraction. The combined layers were evaporated to dryness under a stream of nitrogen at 30°C and the dry residue reconstituted in 1000 µL methanol containing 1% BHT. The liver samples were additionally diluted twofold with the same solution. Finally, 150 µL of each sample was transferred into a round-bottom 96-well polypropylene plate and analyzed.

*LC-MS/MS analysis*

Determination of retinol and $\alpha$-tocopherol in rat serum and tissues was carried out by isotope dilution method using high-performance liquid chromatography coupled with tandem mass spectrometry (LC-MS/MS). LC-MS/MS analysis was performed using a high-performance liquid chromatography (HPLC) system (Shimadzu, Japan) coupled to a QTRAP 4500 triple quadrupole mass spectrometer equipped with Turbo V electrospray ion source (Sciex, Framingham, USA). The HPLC system was equipped with two binary pumps (Exion LC AC, Sciex, Framingham, USA), a degasser (Exion LC, Sciex, Framingham, USA), an autosampler (Eksigent, Sciex, Framingham, USA),

and a column oven (Exion LC AC, Sciex, Framingham, USA). The Kinetex® C18 column (3 mm × 50 mm; 2.6 μm) was introduced for the separation of the analytes. The separation process was conducted at a temperature of 40°C. The samples were injected into the HPLC column at a flow rate of 0.5 mL/min using a gradient of mobile phase A (a mixture of 2 mM ammonium formate and 2 mM formic acid in H2O and B (a mixture of 2 mM ammonium formate and 2 mM formic acid in IPA). The injection volume was 20 μL. The gradient elution program for serum samples was as follows: 0 min–50%B, 4 min–98%B, 5.5 min–98%B, 5.6 min–50%B. The total run time was 7 min. With regard to tissues samples it was as follows: 0 min–50%B, 7 min–92%B, 7.1 min–98%B, 9.1 min–98%B, 9.2 min–50%B with a total run time of 11 min. The injections were carried out with an auto sampler maintained at 8°C. The electrospray ionization (ESI) conditions were as follows: positive ionization mode source voltage 5500V, capillary temperature 600°C, curtain gas pressure 30 psi, nebulizing gas 60 pis, drying gas 60 psi. The ESI parameters were optimized using the instrument-integrated syringe pump analyses provided by the Analyst software package (Analyst 1.6.2). For each of the compounds under investigation, the following parameters were optimized for MS/MS determination: entrance potential (EP), declustering potential (DP), collision cell entrance potential (CEP), collision energy (CE), and collision cell exit potential (CXP). The quantification of analytes was conducted using the selected reaction monitoring mode (SRM). For each analyte, the optimum conditions in SRM mode were determined in infusion mode, and two SRM pairs were chosen as representatives (SRM1 and SRM2) (Table S1). Due to the higher intensity of peak obtained using the SRM1 pairs, they were selected for quantitative analyses. SRM2 pairs were utilized as qualifier transitions for additional confirmation of the analyte presence.

Table S1. LC-MS/MS characteristics in the positive ionization mode.

| **ID†** | **Q1 [m/z]** | **Q3 [m/z]** | **DP [V]** | **EP [V]** | **CE [V]** | **CXP [V]** |
|---|---|---|---|---|---|---|
| $\alpha$-tocopherol 1 | 431.3 | 165.3 | 40 | 10 | 35 | 14 |
| $\alpha$-tocopherol 2 | 431.3 | 69.1 | 40 | 10 | 55 | 14 |
| Retinol 1 | 269.3 | 77.1 | 40 | 10 | 75 | 14 |
| Retinol 2 | 269.3 | 213.2 | 40 | 10 | 75 | 14 |
| $\alpha$-tokoferol-d6 | 437.3 | 171.1 | 40 | 10 | 35 | 14 |
| Retinol-d5 | 274.3 | 77.2 | 40 | 10 | 75 | 14 |

Abbr. in the text. † transitions marked with the number '1' were used as quantifier, transitions marked with the number '2' were used as qualifiers.

Calibration curves for serum samples were generated by spiking ultra-low vitamin E and A serum with the corresponding standard compounds. Stock solutions were prepared by the addition of appropriate amounts of each standard to 1 mL of ultra-low vitamin E and A serum, followed by conditioning at room temperature with gentle swirling for 60 minutes in the dark. Subsequently, the serial dilution of the stock solution was prepared using ultra-low vitamin E and A serum as the diluent to obtain the remaining calibration solutions. However, due to the absence of commercially available lyophilized kidney and liver matrices that are free of vitamins A and E, solvent-based calibration curves were introduced. Given the pronounced differences in dynamic ranges between matrices, independent calibration curves were established, with concentrations adjusted to the expected levels of each analyte in serum and tissue samples. For serum samples, $\alpha$-tocopherol ranged from 1.000 to 20.000 ng/mL and retinol from 50 to 500 ng/mL. For tissue samples, retinol ranged from 0.1–1 μg/g kidney and 20–200 μg/g liver, while $\alpha$-tocopherol ranged from 30–300 μg/g tissue in both

liver and kidney. Each of calibration point was prepared in triplicate in accordance with the standard sample preparation protocols. The coefficients of determination ($R^2$) for the curves were found to be greater than or equal to 0.99. The linearity of the assay was assessed by means of a linear regression with 1/x regression weighting. At the lowest calibration points, accuracies for both $\alpha$-tocopherol and retinol fell within the acceptable range of 80–120%, with signal-to-noise ratios (S/N) reaching 680 and 140 in serum for $\alpha$-tocopherol and retinol, respectively, and 8.240, 50, and 550 in tissue matrices for $\alpha$-tocopherol, kidney retinol, and liver retinol, respectively. In certified reference materials (lyophilized human serum) were included as quality controls in each analytical series. These were reconstituted according to the manufacturer's instructions and subsequently processed in accordance with the same sample preparation protocol as applied to the study samples. Quality controls were analyzed at two concentration levels for each analyte.
