# Supplementary material for: Betaine Alters the Interplay of the Adenosine and NO Systems in the Control of Renal Regional Haemodynamics and Excretion in Diabetic Female Rats
Source: Int J Mol Sci. 2026 May 2;27(9):4076. doi: 10.3390/ijms27094076 (PMC13163498; doi:10.3390/ijms27094076)
Supplement: Supplementary file 1 [file ijms-27-04076-s001.zip › Supplementary file S 2 Results _15-01-2026.pdf]

Supplementary file S 2 Results

Subsection 2.1. Table S1

Subsection 2.2. Vitamins concentration in renal and hepatic tissue

2.1.

Table S1. Plasma (tail vein samples) and the data of 24 h observations in metabolic cages for the samples collected before (day 0) and 10 or 14 days after STZ or solvent injections in female Sprague-Dawley (Tac:Cmd:SD) rats, NG or DM (normoglycaemic or diabetic, respectively): left side - rats pre-treated for 14 days with betaine; right hand side - rats pre-treated for 14 days with betaine and co-treated (+) for the last 4 days with L-NAME (the values shown in bold).

Parameter		Days after buffer or STZ injection Betaine pre-treatment			Days after buffer or STZ injection Betaine + L-NAME pre-treatment		
		0	10	14	0	10	14
Plasma osmolality	NG	307 ± 3	314 ± 5	328 ± 1	305 ± 5	304 ± 6	**309 ± 5**
(mosmol/kg H_2O)	DM	307 ± 4	330 ± 7	324 ± 5	309 ± 2	323 ± 7	**346 ± 13**
Food intake	NG	16 ± 1	14 ± 1	13 ± 1	14 ± 1	13 ± 2	**12 ± 3**
(g/24 h)	DM	16 ± 4	41 ± 6*	31 ± 3*	19 ± 1	17 ± 1	**20 ± 1**
Faeces excretion	NG	8 ± 1	9 ± 1	9 ± 1	6 ± 1	6 ± 1	**5 ± 1**
(g/24 h)	DM	9 ± 2	11 ± 3	14 ± 3*	7 ± 1	7 ± 1	**8 ± 1**
Urine potassium	NG	3.7 ± 0.2	3.6 ± 0.4	3.5 ± 0.5	3.4 ± 0.2	2.4 ± 0.6	**2.5 ± 0.5**
excretion (mmol/24 h)	DM	3.1 ± 0.8	4.6 ± 0.4*	4.8 ± 0.7	4.9 ± 1.0	4.7 ± 0.5	**5.0 ± 0.3**

STZ – streptozotocin; L-NAME (NG-nitro-L-arginine methyl ester, a non-selective nitric oxide synthase inhibitor) and betaine were dissolved in drinking water. * significantly different from day 0.

2.2. Vitamins concentration in renal and hepatic tissue

Table S2 A. Retinol concentration in renal and hepatic tissue harvested in normo- (NG) and hyperglycaemic (DM) female rats without or with betaine (Bet) treatment.

Retinol [µg/g tissue]	NG	DM	NG+Bet	DM+Bet
left kidney	0.69 ± 0.06	0.63 ± 0.12	0.35 ± 0.06	0.54 ± 0.04
right kidney	0.57 ± 0.03	0.87 ± 0.04	0.55 ± 0.02	0.66 ± 0.01
both kidneys	0.63 ± 0.04	0.75 ± 0.08	0.45 ± 0.06 *	0.60 ± 0.03
liver	67.8 ± 8.7	36.5 ± 2.3 *	54.1 ± 13.6	65.2 ± 3.4 #

* significantly different from NG rats; # significantly different from DM rats.

Table S2 B. α-tocopherol concentration in renal and hepatic tissue harvested in normo- (NG) and hyperglycaemic (DM) female rats without or with betaine (Bet) treatment.

α-tocopherol [µg/g tissue]	NG	DM	NG+Bet	DM+Bet
left kidney	92.8 ± 1.0	84.1 ± 1.5	75.3 ± 13.7	67.7 ± 7.7
right kidney	92.9 ± 2.7	84.3 ± 1.6	86.6 ± 16.5	69.0 ± 3.5
both kidneys	92.9 ± 4.6	84.2 ± 1.0	80.9 ± 9.3	68.4 ± 3.8
liver	78.4 ± 11.9 #	161.0 ± 22.7	72.5 ± 4.2	75.3 ± 4.9 #

significantly different from DM rats.
