# Supplementary material for: Betaine Alters the Interplay of the Adenosine and NO Systems in the Control of Renal Regional Haemodynamics and Excretion in Diabetic Female Rats
Source: Int J Mol Sci. 2026 May 2;27(9):4076. doi: 10.3390/ijms27094076 (PMC13163498; doi:10.3390/ijms27094076)
Supplement: Supplementary file 1 [file ijms-27-04076-s001.zip › Supplementary file S 3 Figure S1 diagram.pdf]

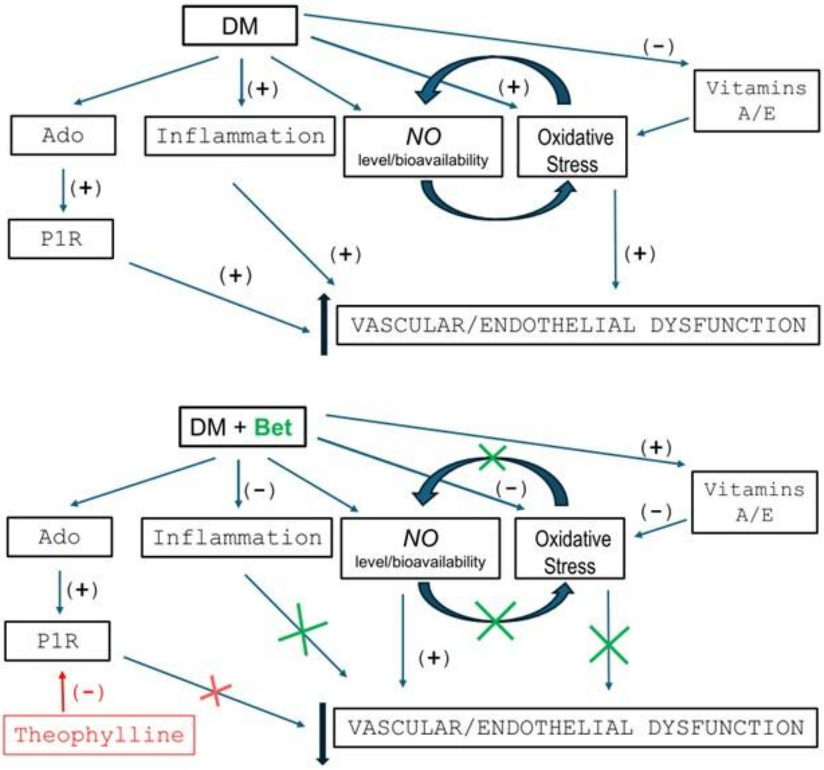

Figure S1. Schematic pathway of the impact of Bet (betaine) intake in diabetes (DM, *diabetes mellitus*) on the factors involved in the vascular/endothelial dysfunction. Ado - adenosine; *NO* - nitric oxide; P1R - purine receptors type P1; Theophylline - non-selective P1R antagonist. 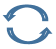 - increased oxidative stress (synthesis of ROS, reactive oxygen species) can interact with *NO* synthesis, which then leads to the increased generation of ROS peroxynitrite, and a decrease in *NO* bioavailability. The potential interplay of P1R and *NO* in diabetes was omitted here to make the diagram more readable.
